# Upregulation of Phosphodiesterase type 5 in the Hyperplastic Prostate

**DOI:** 10.1038/srep17888

**Published:** 2015-12-10

**Authors:** Wenhao Zhang, Ning Zang, Yaoming Jiang, Ping Chen, Xinghuan Wang, Xinhua Zhang

**Affiliations:** 1 Department of Urology, Zhongnan Hospital of Wuhan University, Wuhan 430071, P.R.China; 2 Medical Scientific Research Center of Guangxi Medical University, Nanning 530021, P.R. China; 3 Department of Urology, the First People’s Hospital of Xiaochang, Hubei 432900, P.R.China

## Abstract

Both erectile dysfunction (ED) and lower urinary tract symptoms (LUTS)/benign prostatic hyperplasia (BPH) are common in the aging male. Numerous clinical trials have demonstrated the efficacy and safety of phosphodiesterase type 5 inhibitors (PDE5-Is) for treating LUTS/BPH with/without ED. However, the influence of BPH on prostatic PDE5 expression has never been studied. A testosterone-induced rat model of BPH was developed and human hyperplastic prostate specimens were harvested during cystoprostatectomy. PDE5, nNOS, eNOS and α_1_-adrenoreceptor subtypes (α_1a_ARs, α_1b_ARs and α_1d_ARs) were determined with real-time RT-PCR for rat tissues whilst PDE5 and α_1_-adrenoreceptor subtypes were determined in human samples. PDE5 was further analyzed with Western-blot and histological examination. Serum testosterone was measured with ELISA. The rat BPH model was validated as having a significantly enlarged prostate. PDE5 localized mainly in fibromuscular stroma in prostate. Our data showed a significant and previously undocumented upregulation of PDE5 in both rat and human BPH, along with increased expression of nNOS and α_1d_ARs for rat tissues and α_1a_ARs for human BPH. The upregulation of PDE5 in the hyperplastic prostate could explain the mechanism and contribute to the high effectiveness of PDE5-Is for treating LUTS/BPH. Fibromuscular stroma could be the main target for PDE5-Is within prostate.

Lower urinary tract symptoms (LUTS)/benign prostatic hyperplasia (BPH) are common in aging male. The prevalence of BPH is approximately 40% for men in their fifties and reaches to 90% for men in their eighties or above^1^ and the incidence of LUTS is around 25% for men in their 50 s or older^2^. This disorder is typically characterized by enlargement of the prostate gland, constriction of the urethra, and the emergence of LUTS. Besides prostatectomy, present pharmaceutical treatments for LUTS/BPH are aimed at relieving symptoms and slowing the progression of the disease. Current oral medical treatment options are 1) α-adrenoceptor antagonists (α-blockers, ABs) which reduce urethral resistance by attenuating the tension of smooth muscle (SM) fibers located in the prostate 2) 5α-reductase inhibitors (5ARIs) which are involved in the hormonal control of prostate growth 3) muscarinic receptor antagonists (MRAs) and 4) a “new emerging treatment” phosphodiesterase type 5 inhibitors (PDE5-Is)^3^,^4^. Several clinical studies have demonstrated the efficacy and safety of PDE5-Is in treating LUTS/BPH. We recently performed a systematic review and network meta-analysis including 64 RCTs with 28196 participants comparing the effectiveness of different oral drug therapies for LUTS/BPH^5^. This data showed that among all the drug treatments, PDE5-Is combined with ABs ranked highest in efficacy for decreasing the international prostate symptom score (IPSS) including total score, storage subscore and voiding subscore. PDE5-Is used alone also showed promising efficacy, with the exception of not improving maximum flow rate (Q_max_). However, the mechanisms by which PDE5-Is alleviates LUTS/BPH remains unclear despite the performance of several basic science studies. Recent experimental studies reported a functional role of PDE5 in lower urinary tracts and suggested a potential significance of the PDE5-nitric oxide (NO)/cyclic guanosine monophosphate (cGMP) cell-signaling pathway in the control of urogenital SM^6^,^7^,^8^,^9^. In the current study we used a rat BPH model and human hyperplastic prostate tissue to investigate the expression of genes involved in the major pathways regulating SM tone, with PDE5 emphasized.

## Results

### BPH rat model

**Figure 1:**
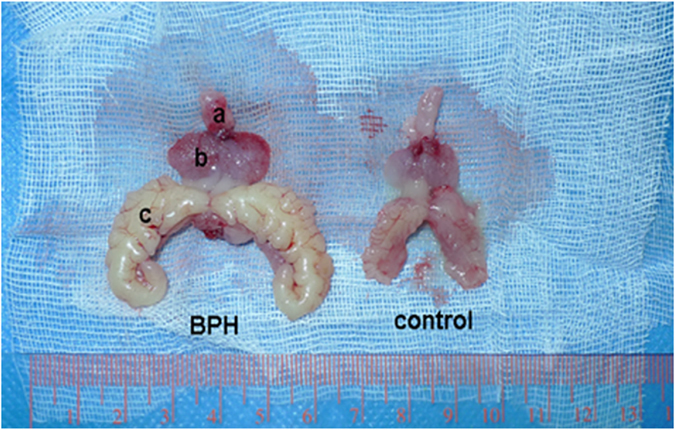
Typical photograph from a BPH and control rat. (a) bladder, (b) ventral prostate, (c) seminal vesicle.

The testosterone (T)-supplementation rat model of BPH was validated through increased weight of the ventral prostate and seminal vesicle (Fig. 1 and Table 1, *P* < 0.001) by 1.6-fold and 2-fold, respectively. Accordingly, T level was found significantly increased for BPH animals (Table 1, *P* *=* 0.016). The body weight of BPH rats was significantly decreased (Table 1, P < 0.001), which may be ascribed to the physiological effect of T. No difference in bladder weight was found between the 2 groups (Table 1). The prostate index [prostate wet weight (mg)/ body weight (g)] was also calculated with a 1.8-fold increase observed in the BPH group (Table 1, *P* < 0.001).

### Histological examination

**Figure 2:**
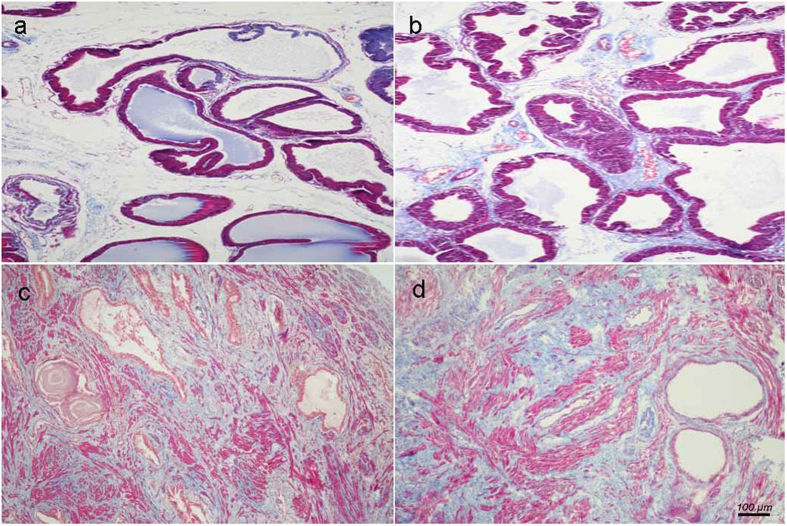
Histological examination of prostate. Masson’s trichrome staining of prostate tissue. Prostatic SM cells were stained red, collagen fibers were stained blue and epithelial cells were stained orange. (**a**) Normal rat prostate. (**b**) BPH rat prostate. Hyperplastic prostate occurred mainly at the epithelial compartment and typical features of glandular hypertrophy was observed including increased acinus number, papillary fronds protruded into the glandular cavities and the epithelial layer thickened. (magnification × 100). (**c**) Normal human prostate. (**d**) Human BPH prostate. An obvious stromal hyperplasia was observed. (magnification × 100, n = 8 for each group).

**Figure 3:**
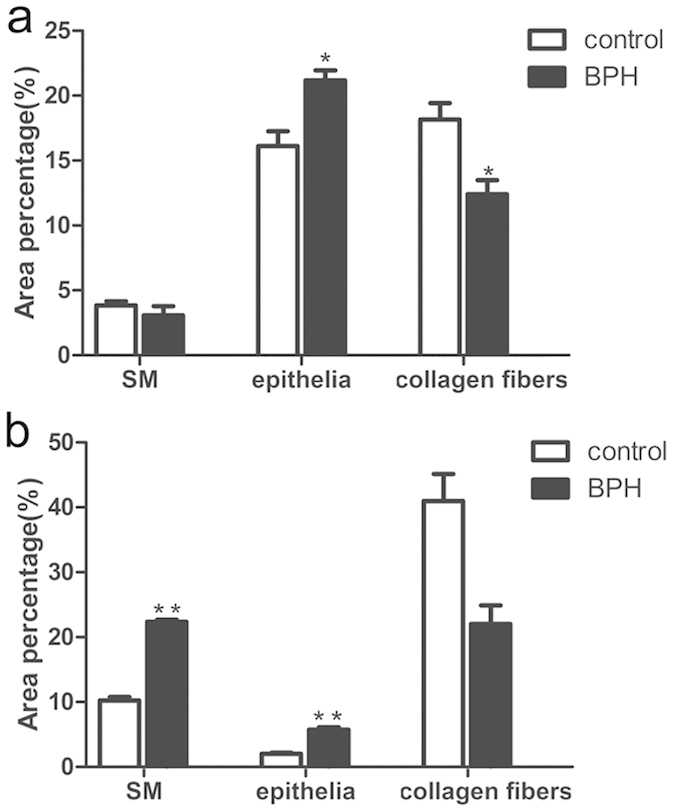
Histological quantification. Area percentage of different component between normal and BPH group. The percentage of each component were quantified from three random 100 × fields of each tissue slices (n = 8 from each group) (**a**) rat prostate. (**b**) human prostate. Boxes, mean; bars, ± S.E.M; **P < 0.01 vs. control; *P < 0.05 vs. control.

Differential histopathology was observed between human and rat BPH by H-E and Masson’s trichrome staining. Specifically, in the rat BPH model, the hyperplasia of the prostate occurred mainly in the epithelium, whereas the stromal component decreased (mainly through the loss of collagen fibers, not SM) (Figs 2a,b and 3a). In contrast, in the human BPH sample, both stroma (mainly through increased SM) and epithelia increased significantly (even though there was a loss of collagen fibers) (Figs 2c,d and 3b).

### Immunostaining

**Figure 4:**
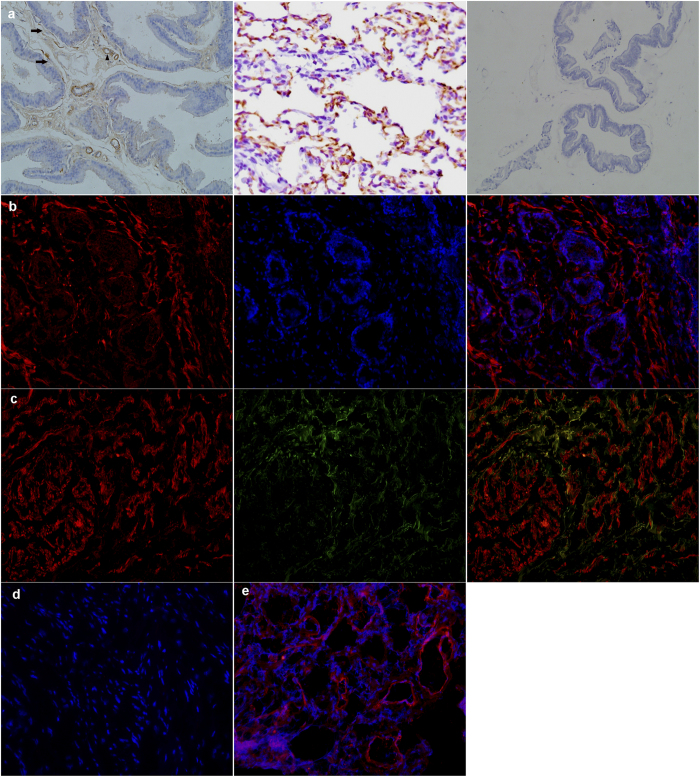
Immunolocalization of PDE5 and nNOS. (**a**) Rat prostate. Left: PDE5 mainly distributed in fibromuscular stroma (black arrows) as well as in the endothelial and smooth muscle cells of blood vessels (black triangle). Middle: rat lung as positive control. Right: negative control. (magnification ×200). (**b**) Human prostate. Left: Cy3-immunofluorescence (red) indicates the PDE5 which was abundantly observed in the fibromuscular stroma. Middle: DAPI (blue) indicates nuclear staining. Right: Merged image. (magnification ×200). (**c**) Double immunofluorescence labeling of PDE5 and nNOS. Left: Cy3-immunofluorescence (red) indicates PDE5. Middle: Cy2-immuno fluorescence (green) indicates nNOS. Right: Merge image indicates the partly colocalization of PDE5 and nNOS in fibromuscular stroma. (magnification ×200). (**d**) Negative control by omitting the primary antibody. (magnification ×200). (**e**) Human lung tissue was used as positive control for PDE5. (magnification ×200, n = 8 from each group).

As shown in Fig. 4, immunohistology demonstrated PDE5 was present in the rat prostate, predominantly in fibromuscular stroma cells and endothelial and SM cells of blood vessels. The human prostate also demonstrated stromal immunolocalization of PDE5. Negative controls omitting the primary antibody failed to stain and positive control using rat and human lung tissue showed a strong immune positivity. Furthermore, the localization of nNOS in human prostate was also investigated, nNOS was partly colocalized with PDE5 in stromal matrix (Fig. 4).

### The mRNA expression of PDE5 and related molecules

**Figure 5:**
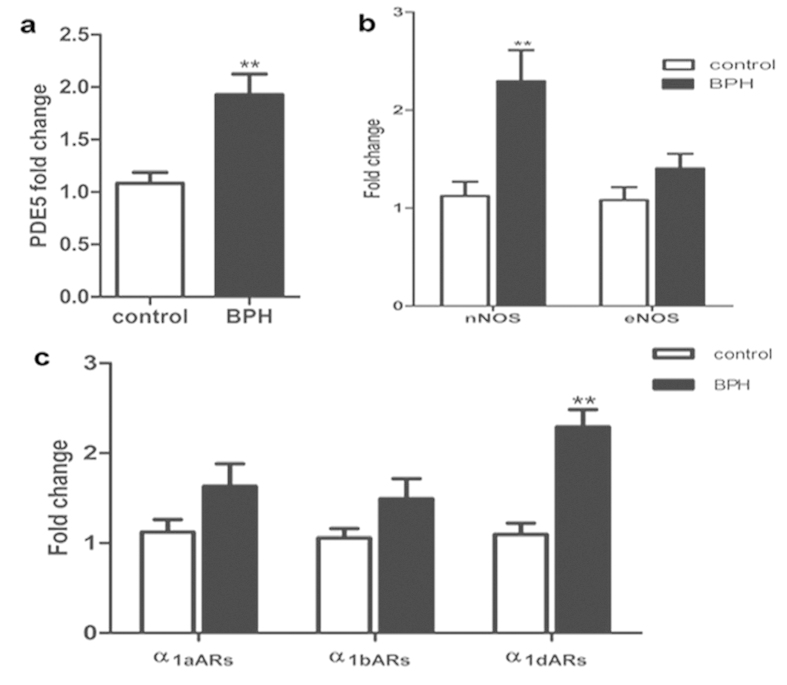
mRNA expression of PDE5, nitric oxide synthase isoforms (nNOS and eNOS) and α_1_-adrenoreceptor subtypes (α_1a_ARs, α_1b_ARs and α_1d_ARs) in rat ventral prostrate (n = 15, 3 replicates per experiment). Boxes, mean; bars, ± S.E.M; **P < 0.01 vs. control; *P < 0.05 vs. control.

**Figure 6:**
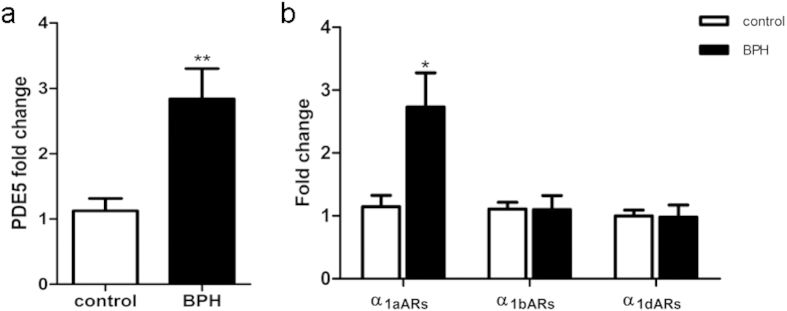
mRNA expression of PDE5 and α_1_-adrenoreceptor subtypes (α_1a_ARs, α_1b_ARs and α_1d_ARs) in human prostate (n = 9, 3 replicates per experiment). Boxes, mean; bars, ± S.E.M; **P < 0.01 vs. control; *P < 0.05 vs. control.

Expression of PDE5, nNOS, eNOS, α_1a_ARs, α_1b_ARs, α_1d_ARs mRNA were determined using quantitative real time RT-PCR in the rat model (Fig. 5). BPH upregulated PDE5 expression by approximately 2-fold at the gene level (*P* *=* 0.001). Real time RT-PCR also showed that nNOS (*P* *=* 0.003) and α_1d_ARs (*P* < 0.001) were augmented significantly in BPH group but with no change in the level of eNOS, α_1a_ARs and α_1b_ARs. The expression of PDE5, α_1a_ARs α_1b_ARs and α_1d_ARs mRNA were also determined in human prostate (Fig. 6). BPH equally upregulated PDE5 and α_1a_ARs expression by 2.5-fold (*P* *=* 0.006*, P* *=* 0.02, respectively) with no change of α_1b_ARs and α_1d_ARs expression.

### The translational expression of PDE5

**Figure 7:**
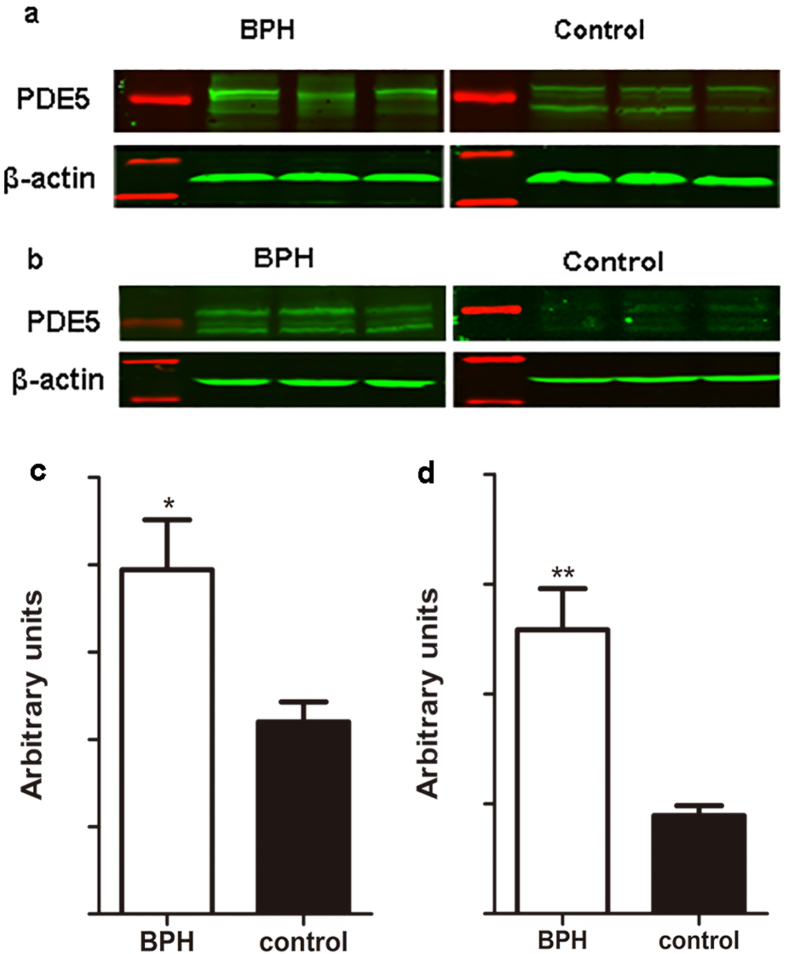
Representative Western blot analysis and relative densitometric quantification of PDE5 in rat and human prostate. (**a**) Representative Western-blot bands of rat prostate. (**b**) Representative Western-blot bands of human prostate. Two major specific bands of the expected sizes (85 and 95 kDa, green) are evident in all lanes, red bands indicates the protein markers. (**c**) Densitometric evaluation of rat prostatic PDE5 expression. (**d**) Densitometric evaluation of human prostatic PDE5 expression. β-actin expression was analyzed as a loading control, results are expressed as ratio of PDE5 in respect to β-actin (n = 10 for rat group, n = 8 for human group, 3 replicates per experiment). Boxes, mean; bars, ± S.E.M; *P < 0.05 vs. control, **P < 0.01 vs. control.

Expression of PDE5 protein was quantified by Western blot analysis (Fig. 7). Two major protein bands were detected at a molecular weight of 85 and 95 kDa, as reported in our previous study^10^. A significant 1.8-fold and over 3-fold increase of PDE5 at translational level was found for BPH rat (*P* *=* 0.013) and human (*P* *<* 0.001), respectively.

## Discussion

Our T induced BPH rat model was validated through hyperplasia of the prostate and seminal vesicle (androgen-sensitive organs) with both organ weights significantly increasing. We observed body weight loss with T supplementation, which may be due to increased daily activity and an increased ratio of lean body mass/fat body mass. In parallel with previous observations^11^,^12^, T injection mainly led to a notable involution of acinar epithelium hyperplasia, such as increased acinus amount, papillary fronds protruding into the glandular cavities and thickening of the epithelial layer while the stromal compartment was relatively decreased. Masson’s trichrome staining further quantified that the BPH epithelia was significantly increased compared with control even though the SM content remained unchanged. In contrast, in the human condition, a predominant stromal hyperplasia was observed with an increase in SM of 2.2-fold.

In both rat and human prostate tissue, PDE5 gene and protein are highly expressed and was immunodistributed exclusively in the stroma. Our finding is in agreement with Fibbi *et al.*^6^ that PDE5 was immunolocalized only in the fibromuscular stroma and vascular (endothelial and SM cells) in the rat and human prostate with no immunoreactivity in the glandular area. However, it contrasts with the findings by Ückert *et al.*^8^ that the glandular and subglandular areas of human prostate also expressed PDE5. This disparity in immunolocalization of PDE5 could be attributed to the different primary antibodies employed as well as the different sources of tissue. In the study of Fibbi *et al.*, prostate tissue was obtained from BPH patients while Ückert *et al.* study obtained tissue from prostate cancer patients. However, Wang *et al.* recently found PDE5 was expressed in both acinar epithelium as well as periacinar SM and lobe-specific PDE5 expression patterns were observed in this study^13^. They speculated that such expression might be associated with glandular secretory function. The stroma-predominant distribution of PDE5 would explain the functional role of PDE5-Is in the control of prostatic SM. In organ bath studies, we and others showed that the exposure of isolated rat or human prostate tissues to PDE5-Is could produce a relaxation of precontracted prostatic strips^10^,^14^. Furthermore, our result of double-immunofluorescent labeling of PDE5 and nNOS from present study showed that these two proteins partly colocalize to prostatic fibromuscular stroma, providing further support for a functional role of PDE5-Is in the NO/cGMP mediated relaxation of prostatic SM.

Importantly, our data for the first time demonstrates an increased expression of PDE5 gene and protein in both the rat and human hyperplastic prostate, with a more significant increase observed in human BPH than in that of rat. We did not perform image quantification for PDE5 immunohistology since the results of quantitative RT-PCR and Western blot are more convincing for quantification. Increased PDE5 expression may be associated with elevated T level. Our findings in the prostate are broadly consistent with other lower urinary tract tissues (corpus cavernosum, bladder and vas deferens) that the expression of PDE5 is T regulated^15^,^16^,^17^,^18^. In our rat model, after 28 days T supplementation, a significant increase in serum T level was detected by ELISA. For human BPH, it is well known that its development is closely associated with androgens and anti-androgen therapy with 5ARIs are effective for larger prostate over 40 ml, although increased estrogen/T ratio seems more important for BPH development^19^. In particular, dihydrotestosterone (DHT) was assumed to play a causative role in the etiology of BPH although this theory still remains speculative and controversial. Previous studies^20^,^21^,^22^ reported levels of intraprostatic DHT increased but these findings were not confirmed by later study^23^, probably due to different methods of tissue retrieval, methods of tissue processing and assays of androgen determination used^24^. In addition, the elevated PDE5 expression may be ascribed to the stromal hyperplasia and increased SM content. Lin *et al.* reported that PDE5 gene is not directly regulated by T and the decreased expression of PDE5 in castrated animals is due to reduced SM content^25^,^26^. Indeed, the stroma-predominant human BPH could contribute to greater PDE5 expression than that of epithelium-predominant rat BPH, as observed in our study. However, in our rat model, histological quantification showed that connective tissue decreased while SM content remained unchanged. Furthermore, in our previous studies on a castrated rat model^10^, the expression of PDE5 was decreased significantly despite a clear relative increase of the stromal layer observed. Therefore, the increased expression of PDE5 observed in our study is more likely to relate to increased gene and protein expression rather than to a change in morphology.

The upregulation of PDE5 in hyperplastic prostate could provide a rationale for the high efficacy of PDE5-Is for treating patients with LUTS/BPH with/without ED. It is well known that prostate enlargement is aging related^2^. In our study, the normal prostate was collected from young brain-dead men with the average age of 29 years old, while BPH specimens were harvested from the elderly with the mean age of 67 years old. Samples included in our study could represent clinical scenarios. We didn’t care the location of tumor in bladder. Since the first clinical trial conducted by Sairam *et al.*^27^ in 2002 with sildenafil for treating LUTS/BPH/ED patients, many clinical trials have been performed demonstrating efficacy and safety of PDE5-Is for treating LUTS/BPH. Our recent systematic review and network meta-analysis^5^ showed that among all the drug treatments, PDE5-Is combined with ABs ranked highest in efficacy for relieving IPSS total score, storage subscore and voiding subscore. PDE5-Is alone also showed promising effect except on Q_max_. Although a number of basic investigations have been done, the mechanisms involved in this treatment are still unclear. To date, a consensus has been reached that the potential mechanisms of PDE5-Is in treating LUTS/BPH are multifactorial^28^: (1) Slight-to-moderate relaxation of muscle tone in prostate and bladder; (2) Significant dilation of local blood vessels which provide adequate blood; (3) Significant augmentation of oxygen perfusion to local organs; (4) Inhibition of afferent nerve activity of bladder; (5) Bluntness of intraprostatic inflammation; (6) Antiproliferation in prostate.

Additionally, expression of other molecular components involved pathways associated with prostatic SM contraction and relaxation were determined in the rat prostate. It was found that several of these genes were differentially modulated by BPH. In rat model, nNOS and α_1d_ARs mRNA were significantly increased with no change for eNOS, α_1a_ARs and α_1b_ARs. Our investigation of α_1_AR subtypes was consistent with Kojima *et al.* study, although a different BPH rat model was used^29^. Also, Hampel *et al.* found that in the lower urinary tract, upregulation of α_1d_ARs correlated with severe bladder hypertrophy and bladder outlet obstruction (BOO)^30^. These results suggested that α_1d_ARs may play a crucial role in rat BPH and BOO. In human, α_1_ARs is richly expressed in the prostate and excessive activity of α_1_-adrenergic SM contraction appears to be a common feature of symptomatic BPH^31^. Moreover, previous quantification of α_1_ARs mRNA expression within human prostate has revealed that α_1a_ARs predominates, followed by α_1d_ARs and α_1b_ARs^32^. This predominance of α_1a_ARs is reportedly more marked in the hyperplastic prostate. Immunohistochemisty showed that the immunoreactivity for α_1a_ARs was exclusively located in the stroma and we consistently found a 2.5-fold increase of α_1a_ARs expression^33^,^34^. The expression of α_1b_ARs and α_1d_ARs were also examined in human prostate and no significant difference was observed. This discrepancy of α_1_-adrenoreceptor subtypes mRNA expression between human and rats BPH could be explained by differences in tissue morphometry. The differential expression and role of different NOS isoforms in the prostate and BPH is interesting and would need to be defined through further experimentation.

In current study, inflammation almost always accompanied BPH. It would be also interesting to further examine the inflammatory factors in prostate and explore whether prostatitis could affect the expression of PDE5 and the efficiency of its inhibitors in BPH/prostatitis patients. In conclusion, our data is the first report demonstrating an increase of PDE5 gene and protein expression both in rat and human hyperplastic prostate. Furthermore, the upregulation of PDE5 in BPH would enhance the efficacy of PDE5-Is within prostate and could therefore explain the possible mechanism and provide rationale for the use of PDE5-Is in treating patients with LUTS/BPH. In addition, fibromuscular stroma cells as well as endothelial and SM cells of blood vessels could be the main target tissue of PDE5-Is in the prostate.

## Materials and Methods

### Animal protocol

A total of 30 specific-pathogen-free (SPF) grade male Wistar rats (12 weeks), weighing 250–300 g, were used. Rats were randomly divided into two groups and fed water and rat chow ad libitum. One group (n = 15) were treated with 0.1 ml sesame oil as controls and the other (n = 15) were subcutaneously injected with 2 mg/d testosterone propionate (Shanghai Tongyong Medical Co., Ltd, Shanghai, China) for 28 days. Rats were weighed and sacrificed under anesthesia using a euthanasia solution containing 10% [w/v] chloral hydrate on day 29. The blood was collected from the abdominal aorta. The whole ventral prostatic lobes, seminal vesicles and bladder were harvested and weighed. Prostatic strips of 1 × 1 × 0.5 cm were put into 10% [v/v] neutral buffered formalin for histological examination and another portion of strips were put into RNA Sample Protector (Takara Bio. Inc.,Otsu, Shiga, Japan) for PCR analysis with the rest of the tissue frozen in liquid nitrogen and saved at −80 °C. All animal experimental procedures were performed strictly in accordance with the care and use of laboratory animals (National Research Council, Washington, DC) and the related ethical regulations of our university.

### Human specimens

Nine human normal prostate samples are obtained from young brain-dead men (mean age, 29.1 ± 1.7 years) undergoing organ donation surgery. Nine human BPH samples are obtained from patients (mean age, 67.7 ± 2.1 years) undergoing cystoprostatectomy for infiltrating bladder cancer without prostate infiltration and LUTS/BPH. Immediately after removal, tissue specimens were shock frozen in liquid nitrogen and stored at −80 °C for subsequent analysis. All tissue samples were obtained after the approval of the Hospital Committee for Investigation in Humans and after receiving written informed consent from all patients or their relatives involved. Human study was conducted in accordance with the principles of the Declaration of Helsinki.

### Serum testosterone level

After blood was collected, serum was then isolated as the supernatant fraction following centrifugation at 3000 g for 10 min. The measurement of T concentrations was via enzyme-linked immunosorbent assay (ELISA) according to the BlueGene Biotech (Shanghai, China) protocol.

### Total RNA extraction and real-time RT-PCR

Total RNA was isolated from the frozen tissues using TaKaRa MiniBEST Universal RNA Extraction Kit (Takara Bio. Inc., Otsu, Shiga, Japan) according to the manufacturer’s protocol. 100 ng of RNA was added in the one-step real-time RT-PCR reaction system (Takara Bio. Inc.,Otsu, Shiga, Japan). The whole system was amplified in a 96-well plate in a 25 μl reaction volume with all samples run in triplicate, using a CFX96 Touch Real-Time PCR Detection System (BioRad, USA). The experimental protocol utilized was first reverse transcription (42 °C 5 min, 95 °C 10 s), followed by an amplification program repeated for 40 cycles (95 °C for 5 s, then 60 °C for 30 s), using SYBR Green measurement. For rat prostate tissue, the following targets were amplified: PDE5, neuronal NOS (nNOS), endothelial NOS (eNOS), and α_1_-adrenoreceptor subtypes (α_1a_ARs, α_1b_ARs and α_1d_ARs), and for human tissue, PDE5, α_1a_ARs, α_1b_ARs and α_1d_ARs were investigated. Primer sequences are shown in Table 2. For relative quantification, gene expression was normalized to expression of β-actin housekeeping gene and compared by 2^−ΔΔCT^ method.

### SDS-PAGE and Western blot analysis

Proteins were extracted from frozen samples using the CelLytic™ NuCLEAR™ Extraction Kit (Sigma-Aldrich, Saint Louis, USA) and 100 μg of each sample was eletrophoresed on a 10% sodium dodecyl sulfate-polyacrylamide (SDS-PAGE) gel (Wuhan Boster Biological Technology Ltd, Wuhan, China) and transferred to polyvinylidene fluoride (PVDF) membrane (Millipore, Billerica, MA, USA) using a Bio-Rad wet transfer system. The membrane was blocked for 2 h at room temperature with Tris-buffered saline with 0.1% [v/v] Tween (TBST) containing 5% [w/v] non-fat dry milk solution. The membrane was incubated overnight with primary PDE5 antibody (Rabbit polyclonal to PDE5A, ab64179) at dilution of 1:1000 (Abcam, Cambridge, UK). Membranes were washed with TBST three times and incubated at room temperature for 1 h with an IRDye 800CW conjugated goat anti-rabbit IgG (LI-COR, Lincoln, USA) at dilutions of 1:15000. After washing, the blots were visualized by scanning using Li-Cor Odyssey Imager (Li-Cor, Lincoln, USA). The bands were quantified by densitometry using Li-Cor Odyssey software. A polyclonal rabbit antibody against β-actin (1:2000; Abcam) was used as a control to ascertain equivalent loading.

### H&E staining and Masson’s trichrome staining

Rat and human prostate tissues fixed in 10% [v/v] neutral buffered formalin for 24–36 h were processed routinely for paraffin embedding. The paraffin-embedded tissue sections (4 μM) were stained with hematoxylin and eosin using standard techniques. The paraffin sections were deparaffinized in xylene followed by graded alcohols. Masson composite staining solution (Fuzhou Maxim Biotech Co., Ltd., Fuzhou, China) was added dropwise for 10 min. The sections were subsequently washed with distilled water, differentiated in phosphomolybdic-phosphotungstic acid solution for 10 min, and incubated with blue staining solution for 5–10 min. Rinsed briefly in distilled water and differentiated in 1% acetic acid solution for 2 minutes. After dehydrated quickly through 95% alcohol, absolute alcohol, the sections were cemented using neutral gum for observation. Using this procedure, prostatic stroma SM cells were stained red, collagen fibers were stained blue and epithelial cells were stained orange. In each sample, we analyzed three areas under magnification (×100). The area percentage of SM, collagen fibers and glandular epithelium were quantitated with Image pro plus 5.0, respectively.

### Immunohistochemistry

Sections for immunohistochemistry were deparaffinized in xylene followed by graded alcohols. Antigen retrieval was performed in 10 mM sodium citrate buffer (pH 6.0) and heated to 96 °C for 3 min. Endogenous peroxidase activity was blocked by using 3% [v/v] H_2_O_2_ solution in methanol at room temperature for 10 min. Sections were incubated with 15% [v/v] normal goat serum for 1 h at 37 °C to block nonspecific binding. 100 μl appropriately diluted PDE5A primary antibody (1:100) was applied to the sections on the slides and incubated in a humidified chamber at 4 °C overnight. Then the sections were stained by routine immunohistochemistry methods. Negative controls were performed for all samples by omitting the primary antibodies. Rat lung tissue was used as a positive control for PDE5A staining. All the stained sections were imaged using Olympus-DP72 light microscope (Olympus, Japan).

### Immunofluroscence

Human prostate were embedded in Tissue-Tec OCT compound (SakuraFinetek Japan, Tokyo, Japan) and snap frozen. Then tissue was sectioned in 10 μM thick slices and thaw, mounted onto glass slides using a cryostat (Leica CM 1850, Wetzlar, Germany), air-dried, and fixed for 10 min in ice-cold acetone. Slides were washed in PBS and then incubated for 2 h in a mixture of PBS supplemented with 0.2% [v/v] Triton X-100 and 0.1% [w/v] bovine serum albumin, followed by incubation overnight with the primary antibody (rabbit polyclonal to PDE5A, 1:100) and antibody mixture of the PDE5A antibody (1:100) and goat polyclonal to nNOS (1:50, Abcam, ab1376 Cambridge, UK). The secondary antibodies employed to visualize the localization of the two primary antibodies (Jackson ImmunoResearch Inc. West Grove, PA, USA) were Cy3-conjugated goat anti-rabbit IgG (1:1000) and Cy2-conjugated donkey anti-goat IgG (1:400). DAPI was used for staining the nucleus. Negative controls were performed for all samples by omitting the primary antibodies. Human lung tissue was used as a positive control for PDE5A staining. Visualization was done with a laser microscope (Olympus, Tokyo, Japan). Colocalization analysis was performed using NIS-Elements Viewer 3.20 (Nikon, Japan).

All experimental protocol were approved by the research committee of Wuhan University.

### Statistical analysis

Results are expressed as the mean ± SD in table and mean ± SEM in bar graph. Student’s t test was used to analyze the differences between two groups. Statistical analyses were performed using GraphPad Prism v5.0 (La Jolla, CA). A *P* < 0.05 was considered statistically significant.

### Ethical standards

All procedures performed in studies involving human participants were in accordance with the ethical standards of the research committee of Wuhan University and the principles of the Declaration of Helsinki. All participants gave written informed consent before taking part in the study.

All procedures performed in studies involving animals were in accordance with the care and use of laboratory animals (National Research Council, Washington, DC) and the ethical standards of Wuhan University.

All experimental protocol were approved by the research committee of Wuhan University.

## Additional Information

**How to cite this article**: Zhang, W. *et al.* Upregulation of Phosphodiesterase type 5 in the Hyperplastic Prostate. *Sci. Rep.* **5**, 17888; doi: 10.1038/srep17888 (2015).

## Figures and Tables

**Table 1:** Variation of biometric and physiological parameters in control and BPH rats.

Group	Body weight (g)		Ventral prostate weight (mg)	Seminal vesicles weight (mg)	Prostate index	T level (ng/mL)	Bladder weight (mg)
	Initial	Final					
Control	274.3(13.1)	429.8(32.7)	778.3(135.3)	1001.8(288.5)	1.8(0.3)	4.4(0.4)	151.2(17.9)
BPH	272.6(12.7)	387.2(13.7)**	1231.3(131.4)**	2483.9(360.6)**	3.2(0.3)**	6.2(0.5)*	147.4(17.3)
P value	0.706	0.000	0.000	0.000	0.000	0.016	0.560

*P* values calculated by unpaired t test. Data are mean ± SD. ^**^P < 0.01 VS control, ^*^P *<* 0.05 VS control.

**Table 2:**
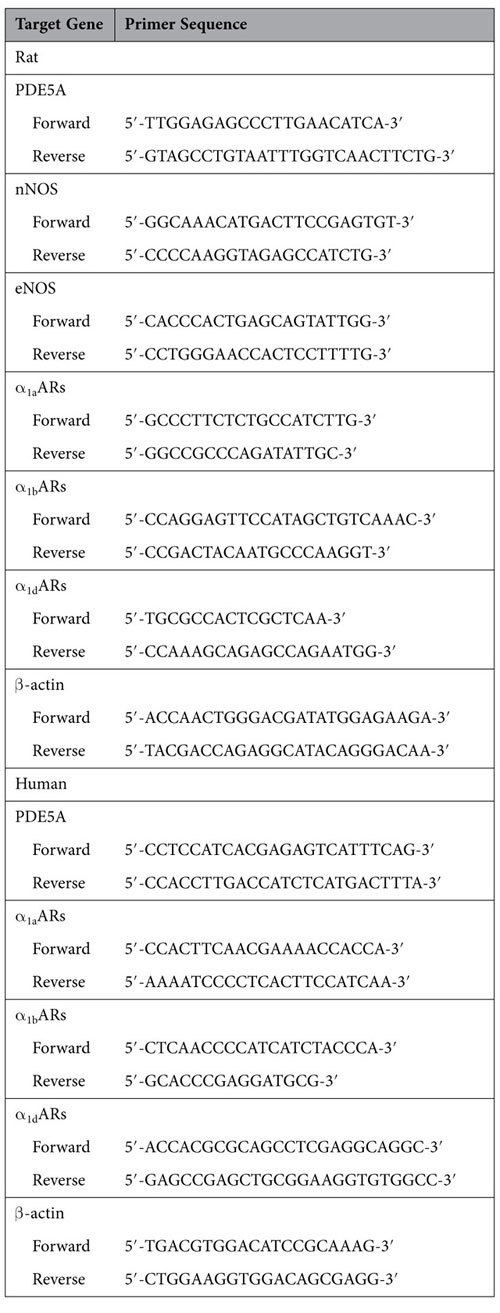
Primer sequences used to amplify target genes by real-time RT-PCR.
